# Intermittent Hypoxia Is Associated With High Hypoxia Inducible Factor-1α but Not High Vascular Endothelial Growth Factor Cell Expression in Tumors of Cutaneous Melanoma Patients

**DOI:** 10.3389/fneur.2018.00272

**Published:** 2018-04-26

**Authors:** Isaac Almendros, Miguel Ángel Martínez-García, Francisco Campos-Rodríguez, Erica Riveiro-Falkenbach, José L. Rodríguez-Peralto, Eduardo Nagore, Antonio Martorell-Calatayud, Luis Hernández Blasco, Jose Bañuls Roca, Eusebi Chiner Vives, Alicia Sánchez-de-la-Torre, Jorge Abad-Capa, Josep Maria Montserrat, Amalia Pérez-Gil, Valentín Cabriada-Nuño, Irene Cano-Pumarega, Jaime Corral-Peñafiel, Trinidad Diaz-Cambriles, Olga Mediano, Joan Dalmau-Arias, Ramon Farré, David Gozal, On Behalf of the Spanish Sleep Network, Elidia Molina Herrera

**Affiliations:** ^1^Unitat de Biofísica i Bioenginyeria, Facultat de Medicina i Ciències de la Salut, Universitat de Barcelona, Barcelona, Spain; ^2^Centro de Investigación Biomédica en Red de Enfermedades Respiratorias (CIBERES), Madrid, Spain; ^3^Institut d’Investigacions Biomèdiques August Pi i Sunyer (IDIBAPS), Barcelona, Spain; ^4^Respiratory Department, Hospital Universitario y Politécnico la Fe, Valencia, Spain; ^5^Respiratory Department, Hospital Universitario de Valme, Seville, Spain; ^6^Pathology Department, Medical School, Universidad Complutense, Instituto i + 12, Hospital Universitario 12 de Octubre, CIBERONC, Madrid, Spain; ^7^Dermatology Department, Instituto Valenciano de Oncología, Valencia, Spain; ^8^Dermatology Department, Hospital de Manises, Valencia, Spain; ^9^Respiratory Department, ISABIAL, Hospital Gral, Univ. Alicante, Alicante, Spain; ^10^Departamento Medicina Clinica, Univ. Miguel Hernandez, Elche, Spain; ^11^Respiratory Department, Hospital san Juan de Alicante, Alicante, Spain; ^12^Respiratory Department, Group of Translational Research in Respiratory Medicine, Hospital Universitari Arnau de Vilanova and Santa Maria, IRBLleida, Lleida, Spain; ^13^Respiratory Department, Hospital Germans Trias i Pujol, Centro de investigacion Biomedica, Madrid, Spain; ^14^Respiratory Department, Hospital Clinic-IDIBAPS, Barcelona, Spain; ^15^Dermatology Department, Hospital de Valme, Seville, Spain; ^16^Respiratory Department, Hospital Universitario Cruces, Bilbao, Spain; ^17^Respiratory Department, Hospital Universitario de Getafe, Madrid, Spain; ^18^Respiratory Department, Hospital Universitario S. Pedro Alcántara, Cáceres, Spain; ^19^Respiratory Department, Hospital 12 de Octubre, Madrid, Spain; ^20^Respiratory Department, Hospital Universitario de Guadalajara, CIBER de enfermedades respiratorias, Madrid, Spain; ^21^Dermatology Department, Hospital de la Santa Creu i Sant Pau, Barcelona, Spain; ^22^Department of Pediatrics, Pritzker School of Medicine, Biological Sciences Division, The University of Chicago, Chicago, IL, United States

**Keywords:** melanoma, intermittent hypoxia, obstructive sleep apnea, hypoxia-inducible factor, vascular endothelial growth factor

## Abstract

Epidemiological associations linking between obstructive sleep apnea and poorer solid malignant tumor outcomes have recently emerged. Putative pathways proposed to explain that these associations have included enhanced hypoxia inducible factor (HIF)-1α and vascular endothelial growth factor (VEGF) cell expression in the tumor and altered immune functions *via* intermittent hypoxia (IH). Here, we examined relationships between HIF-1α and VEGF expression and nocturnal IH in cutaneous melanoma (CM) tumor samples. Prospectively recruited patients with CM tumor samples were included and underwent overnight polygraphy. General clinical features, apnea–hypopnea index (AHI), desaturation index (DI4%), and CM characteristics were recorded. Histochemical assessments of VEGF and HIF-1α were performed, and the percentage of positive cells (0, <25, 25–50, 51–75, >75%) was blindly tabulated for VEGF expression, and as 0, 0–5.9, 6.0–10.0, >10.0% for HIF-1α expression, respectively. Cases with HIF-1α expression >6% (high expression) were compared with those <6%, and VEGF expression >75% of cells was compared with those with <75%. 376 patients were included. High expression of VEGF and HIF-1α were seen in 88.8 and 4.2% of samples, respectively. High expression of VEGF was only associated with increasing age. However, high expression of HIF-1α was significantly associated with age, Breslow index, AHI, and DI4%. Logistic regression showed that DI4% [OR 1.03 (95% CI: 1.01–1.06)] and Breslow index [OR 1.28 (95% CI: 1.18–1.46)], but not AHI, remained independently associated with the presence of high HIF-1α expression. Thus, IH emerges as an independent risk factor for higher HIF-1α expression in CM tumors and is inferentially linked to worse clinical CM prognostic indicators.

## Introduction

Cutaneous melanoma (CM) is a very aggressive type of skin cancer that is not only fraught with a high mortality rate, but is also a cancer type whose incidence has continued to increase over the last several decades (1). In an effort to identify tumors with more aggressive properties, several investigators have postulated that the high proliferative rates of CM could induce the presence of episodic intra-tumoral hypoxia, which in turn could foster alterations in melanoma malignant cells to promote invasion and metastasis, as well as resistance to chemotherapy (2–4).

Since intermittent hypoxia (IH)-induced alterations in tumor malignancy are driven, at least in part, by the transcriptional activity of hypoxia inducible factor-1α (HIF-1α) pathways (5–7), it is not surprising that many of the HIF-1α genes that are involved in regulation of cellular bioenergetics, angiogenesis, and expansion of the vascular supply network exhibit increased expression within rapidly proliferating tumors and are, therefore, considered as potentially viable reporters of CM aggressiveness and poor outcomes (8, 9). In addition, one of the major genes regulated by HIF-1α transcriptional activity is vascular endothelial growth factor (VEGF), a major regulator of the number of capillaries within a tissue (10, 11) and is also active in a vasculogenic mimicry capacity (12). Studies exploring the expression HIF-1α and VEGF in CM have, however, resulted in somewhat inconsistent results, with a propensity of the findings supporting the concept that increased expression of HIF-1α and VEGF in CM is associated with tumor staging as well as with other prognostic indicators (13–21).

Obstructive sleep apnea (OSA) is a highly prevalent disorder characterized by the presence of increased upper airway collapsibility during sleep that leads to either upper airway collapse or restricted airflow and consequent periodic hypoxia and hypercapnia events, usually terminated by arousal and restoration of airflow (22–24). The cyclical hypoxic events that characterize OSA have been implicated in a vast array of OSA-associated morbidities involving cardiovascular, metabolic, and cognitive systems (25–27). In recent years, potential associations between OSA and cancer have been reported and primarily ascribed to the effects of IH on tumor biology (28–31). In addition, both experimental and clinical evidence has indicated that CM is one of the more susceptible tumors to the IH patterns that characterize OSA (32–34).

Based on aforementioned considerations, we hypothesized that semiquantitative assessments of HIF-1α and VEGF immunoreactivity in excised CM tumors may be associated with typical severity measures of OSA. Hence, the objective of the present study was to analyze potential associations between the expression of HIF-1α and VEGF and measures of respiratory disturbance during sleep in a large prospective series of patients suffering from CM.

## Materials and Methods

### Design, Setting, and Patients

We conducted a prospective, multicenter study in 29 Spanish University Hospitals. Consecutive patients diagnosed with CM by the dermatology or oncology departments were eligible for inclusion in this study. Patients were excluded if they had melanoma of an unknown primary cause, melanoma in mucosa or melanomas “*in situ*,” respiratory failure, heart failure grades III–IV NYHA, pregnancy, or a prior diagnosis of SDB or CPAP treatment. The study was approved by the Ethical Committee of each participating center, and all patients signed the informed consent.

### Procedures

All tumors were surgically resected and subjected to clinical staging. In addition to standard pathologic assessment in each center to establish the diagnosis of CM, all the specimens were re-evaluated in a melanoma reference center by an expert pathology panel whose members were blinded to any of the immunostaining findings. For each tumor, the following pathologic features were assessed by the panel: Breslow tumor thickness (in millimeter, and categorized as ≤1.00, 1.01–2.00, 2.01–4.00, and >4.00), ulceration (presence vs. absence), tumor mitotic rate (according to “hot spot” method and categorized in: >5 vs. ≤5 mitoses/mm^2^), and regression (presence vs. absence). Furthermore, the following clinical characteristics were also documented: tumor location (head/neck, upper extremities, trunk, lower extremities, acral), age at diagnosis, sex, body mass index (BMI, kg/m^2^), skin phototype, and stage at diagnosis [localized (I–II), loco-regional disease (III), and distant metastasis (IV)] by the treating dermatologists and institutional pathologists. In addition, the staging of each tumor was confirmed by a multi-institutional tumor board that was unaware of the immunohistological findings described below. Other markers of melanoma aggressiveness, namely, the presence of ulceration and regression in biopsy tissue, histologic subtypes of melanoma, presence of metastasis, and the invasion of sentinel lymph nodes were also documented.

### Immunohistochemistry

**Figure 1:**
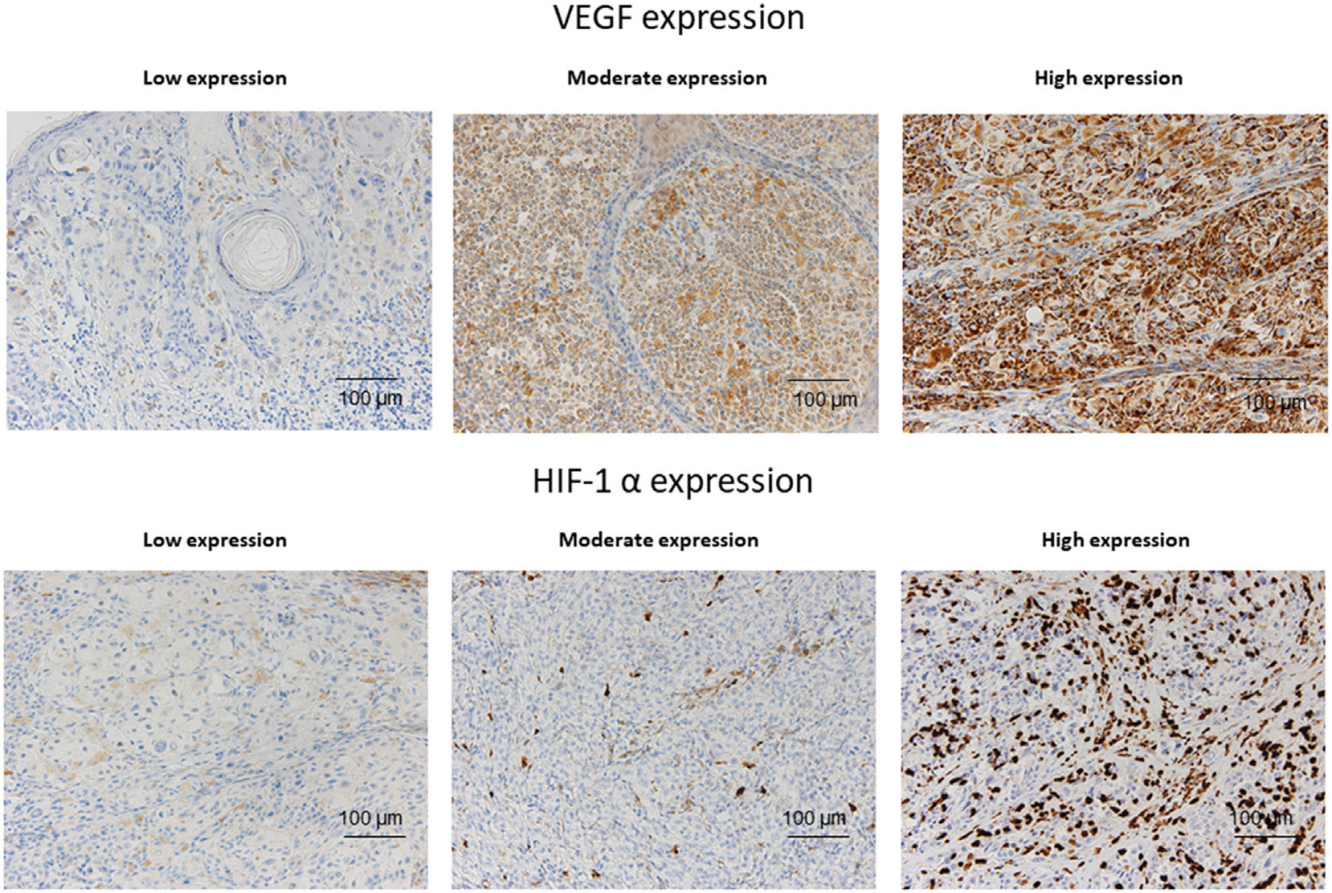
Representative examples of VEGF and HIF-1α expression according to expression levels (low, medium, and high) in melanoma sections.

Immunohistochemistry was performed on 4-μm-thick sections of formalin-fixed, paraffin-embedded melanoma samples. Immunostaining was performed on a Leica Bond-III stainer (Leica Biosystems, Newcastle, UK) using the Leica Bond Polymer Refine Kit (Leica Biosystems). Melanoma samples were stained with a 1:2 dilution of anti-VEGF prediluted antibody (ab27620, Abcam, Cambridge, UK) and with a 1:100 dilution of anti-HIF-1α antibody (ab51608, Abcam). The staining results were independently analyzed by two expert pathologists who were blinded to the staging and clinical features of the subjects. VEGF-specific staining was scored taking into account both the percentage of positive cells (0, <25, 25–50, 51–75, >75%) and expression intensity (low, moderate, or high) (Figure 1). HIF-1α-specific nuclear staining was scored taking into account the percentage of positive cells (0, 0–5, 6–10, >10%) as well as the expression intensity (low, moderate, or high) (Figure 1). After the blind scoring of immunohistochemical stains in each tumor was completed, the data were tabulated, and are presented accordingly.

### Sleep Study

All patients included in the study underwent a diagnostic sleep study by means of respiratory polygraphy, using a device validated against polysomnography (Embletta Gold in 37% of the patients and Alice PDX in 63% of patients) both validated against PSG, and following the Spanish Society of Pneumology and Thoracic Surgery Guidelines for OSA diagnosis and treatment. The time span between the initial appointment for suspicion of melanoma and the sleep study was always lower than 6 months. Every sleep study was manually scored by skilled staff. All the studies included continuous recording of the oro-nasal flow and pressure, respiratory movements, and oxyhemoglobin saturation (SpO_2_). Apnea was defined as complete cessation of oro-nasal flow for ≥10 s and was classified as either obstructive or central, based on the presence or absence of respiratory efforts. Hypopnea was defined as a 30–90% reduction in oro-nasal flow for ≥10 s followed by a ≥3% decrease in SpO_2_, while Tsat90 was defined as the percentage of recording time with SpO_2_ <90%. The recording time, desaturation indices at 4% and the mean baseline nocturnal, baseline daytime, and minimum nocturnal saturation were also recorded. Those tests in which the patients claimed to sleep less than 4 h, or in which there were less than 5 h of nocturnal recording, were repeated.

### Statistical Analysis

The SPSS 20.0 statistical package (Chicago, IL, USA) was used for all analyses. The data were expressed by using the mean and SD, or the median and the interquartile range for quantitative variables, according to whether or not they followed a normal distribution. The qualitative and dichotomic variables were expressed as the absolute value and the percentage with respect to the total. For analyses purposes, histochemical assessments of HIF-1α and VEGF expression variables were dichotomized: HIF-1α expression >6% (high expression) was compared with those samples with <6%, and VEGF expression >75% of cells was compared with those with <75%.

The comparison between these two groups’ baseline variables was made with the Student’s *t*-test for independent means or the Mann–Whitney test, according to the distribution of the quantitative variables, while the chi-square test was used to compare the two percentages. The factors independently related to the presence of a high HIF-1α or VEGF expressions were determined by introducing into two multivariate logistical regression analyses those variables which, in the opinion of the researchers, could have clinical importance. Odds ratios with 95% confidence intervals were calculated for each molecule expression. Although a two-tailed *p*-value of <0.05 was initially considered as indicative of statistical significance, Bonferroni test was used to correct *p*-values for multiple comparisons as appropriate.

## Results

### Baseline Characteristics

Of 476 eligible patients, 443 were prospectively recruited and provided informed consent to participate in this study and finally, 376 subjects had biopsy tissues available for analysis. The baseline clinical and tumor characteristics are depicted in Table 1, and the sleep-related symptoms and sleep test variables are shown in Table 2. The patients had a mean age of 56.4 (15.1) years (52.9% males), BMI of 27.3 (4.6) kg/m^2^, apnea–hypopnea index (AHI) of 14.9 (16.5) event/h, and DI4% of 10.2 (13.7) desaturations/h. Regarding the melanoma tumor, the mean Breslow index was 1.70 (2.5) mm, the most common anatomical site was the body (41.8%), and only four patients had distant metastasis at the time of the diagnosis.

**Table 1:** Patient and cutaneous melanoma characteristics (*n* = 376).

Parameters	*N* (%)
Patients, *n* (%)	376 (100%)
Gender, *n* (% males)	199 (52.9%)
Age (years)	56.4 (15.1)
BMI, kg/m^2^	27.3 (4.6)
**Skin phototype *n* (%)**	
I	13 (3.5%)
II	182 (48.4%)
III	159 (42.3%)
IV	22 (5.9%)
V	0 (0%)
Melanoma family history, *n* (%)	37 (9.8%)
Sun exposure >20 h/week, *n* (%)	121 (32.2%)
Breslow index (thickness in mm)	1.70 (2.5)
Ulceration, *n* (%)	65 (17.3%)
Regression, *n* (%)	91 (24.2%)
Mitotic rate (>5 mitotic cells/mm^2^), *n* (%)	55 (14.6%)
**Clark level, *n* (%)**	
I	2 (0.5%)
II	133 (35.4%)
III	130 (34.6%)
IV	99 (26.3%)
V	10 (2.7%)
**Subtype, *n* (%)**	
Superficial spreading melanoma	268 (71.3%)
Nodular melanoma	60 (16%)
Lentigo malignant melanoma	24 (6.4%)
Acral lentiginous melanoma	19 (5.1%)
Sentinel lymph node, *n* (%)	43 (11.4%)
**Anatomical site, *n* (%)**	
Head and neck	54 (14.4%)
Body	157 (41.8%)
Upper limb	55 (14.6%)
Lower limb	94 (25%)
Acral	15 (4%)
**Extension, *n* (%)**	
Local (I–II)	324 (86.2%)
Loco-regional (III)	42 (12.2%)
Distant metastasis (IV)	4 (0.9%)
Previous nevus, *n* (%)	104 (27.7%)

**Table 2:** Sleep characteristics and other comorbidities.

Parameters	*N* (%)
**Site of sleep study, *n* (%)**	
Hospital	110 (29.3%)
Home	266 (70.7%)
Sleep study time, h	7.2 (1.2)
Chronic snoring (at least 3 day/week), *n* (%)	241 (64.1%)
Number of days/week	3.7 (2.96)
Witnessed apnea, *n* (%)	75 (19.9%)
Epworth score	6 (3.5)
Epworth ≥ 10	59 (15.7%)
Neck circumference, cm	37.9 (4.5)
Sleep duration, h	7.4 (1.27)
<6 h	77 (20.5%)
6–8 h	239 (63.6%)
>8 h	60 (16%)
Insomnia	30 (8%)
Baseline SpO_2_	97 (3.3)
Apnea–hypopnea index (AHI), events/h	14.5 (16.2)
AHI ≥ 5	247 (66%)
AHI ≥ 15	128 (34.1%)
AHI ≥ 30	54 (14.4%)
Central AHI, events/h	1.1 (3.8)
DI4%, desaturations/h	10.5 (13.5)
DI3%, desaturation/h	15.9 (18.7)
Nadir SpO_2_	83.6 (8.96)
Nocturnal average SpO_2_	93.6 (3.84)
Tsat90%	6.1 (12.6)

*Quantitative variables are tabulated as mean (SD)*.

### VEGF and HIF-1α Expression

The vast majority of melanoma samples presented high VEGF expression (333 patients, 88.8%), and non-high HIF-1α expression (360 patients, 95.7%). Only two samples (0.5%) presented no VEGF expression, and only 16 samples (4.2%) presented high HIF-1α expression, with only five patients presenting HIF-1α expression in more than 10% of cells. 6 (1.6%), 12 (3.4%), and 24 (6.4%) samples presented VEGF expression in less than 25%, between 25 and 50%, and between 51 and 75% of cells, respectively.

### Univariate Analysis

Those patients with CM and high VEGF expression were most frequently females and showed significantly less mitotic index (Table 3), higher AHI value (nearly statistically significant), and lower Tsat90 (Table 4).

**Table 3:** Comparisons between expression of vascular endothelial growth factor (VEGF) and general characteristics and aggressiveness markers of melanoma.

Variable	VEGF high expression (*n* = 334)	VEGF non-high expression (*n* = 42)	*p*-Value
Age (years)	56.3 (15.3)	57.4 (13.9)	0.21
BMI (kg/m^2^)	27.2 (4.6)	29.1 (3.8)	0.224
Gender (% males)	51%	62%	**0.0001**
Breslow index	1.67 (2.6)	1.89 (2.26)	0.59
Ulceration	17%	21%	0.19
Positive sentinel lymph	12%	12%	0.92
Mitotic index (/mm^2^)	2.19 (3.7)	3.46 (6.6)	**0.006**
Clark index	2.94 (0.86)	3.1 (0.85)	0.91
Regression	24%	24%	0.90

*Bold fond indicates p < 0.05*.

**Table 4:** Comparisons between expression groups of vascular endothelial growth factor (VEGF) regarding sleep-disordered breathing variables and intermittent/continuous hypoxia indicators.

Variable	VEGF high expression (*n* = 334)	VEGF non-high expression (*n* = 42)	*p*-Value
Apnea–hypopnea index (/h)	14.6 (16)	15.8 (20)	0.058
DI4% (/h)	10 (13.2)	10.9 (16.6)	0.24
Mean SpO_2_	93.8 (1.97)	93.3 (2.08)	0.69
Nadir SpO_2_	83.5 (9.56)	83.8 (6.8)	0.38
Tsat <90%	5.4 (12.03)	8.68 (15.7)	**0.012**
Epworth score	6.12 (3.5)	5.7 (3.2)	0.67

*Bold fond indicates p < 0.05*.

On the other hand, those patients with CM and high HIF-1α expression showed significantly more CM aggressiveness markers, including increased mitotic index, higher Breslow index, the presence of ulceration and proportion of positive sentinel nodes (Table 5), and greater SDB severity regarding their corresponding DI4% (near statistically significant association: *p* = 0.08) and nadir SpO_2_ values (Table 6).

**Table 5:** Comparisons between the groups with high and low expression of hypoxia inducible factor (HIF)-1α regarding their general characteristics and aggressiveness markers of melanoma.

Variable	HIF-1α high expression (*n* = 16)	HIF-1α non-high expression (*n* = 360)	*p*-Value
Age (years)	62.3 (15)	56.2 (15)	0.61
BMI (kg/m^2^)	27.8 (4.7)	27.3 (4.6)	0.86
Gender (% males)	56%	53%	0.38
Breslow index	5.53 (4.8)	1.53 (2.21)	**0.0001**
Ulceration	44%	16%	**0.0001**
Positive sentinel lymph	40%	10%	**0.0001**
Mitotic index (/mm^2^)	6.44 (7.9)	2.1 (3.8)	**0.01**
Clark index	3.7 (0.79)	2.9 (0.85)	0.65
Regression	25%	24%	0.90

*Bold fond indicates p < 0.05*.

**Table 6:** Comparisons between the groups with high and low expression of hypoxia inducible factor (HIF)-1α in relation to sleep-disordered breathing variables and intermittent/continuous hypoxia indicators.

Variable	HIF-1α high expression (*n* = 16)	HIF-1α non-high expression (*n* = 360)	*p*-Value
Apnea–hypopnea index (/h)	24.1 (17.8)	14.5 (16.3)	0.37
DI4% (/h)	18.8 (16.6)	9.8 (13.5)	0.081
Mean SpO_2_	93.5 (1.41)	93.8 (1.9)	0.153
Nadir SpO_2_	79.1 (12.8)	83.7 (9.1)	**0.008**
Tsat <90%	5.2 (6.8)	5.85 (12.7)	0.329
Epworth score	6.6 (3.3)	6.1 (3.6)	0.88

*Bold fond indicates p < 0.05*.

### Multivariate Analysis: Logistic Regressions

To explore which variables were independently associated with high VEGF expression, four variables were introduced in the stepwise logistic regression: gender, AHI, Tsat90%, and mitotic index. However, none of these variables were significantly associated with VEGF expression (Table 7).

**Table 7:** Logistic regression analysis illustrating the lack of independent relationships between VEGF expression and melanoma aggressiveness, gender, and sleep study variables.

Variable	β coefficient	SE	Odds ratio	95% CI	*p*-Value
Gender	0.21	0.329	1.24	0.65–2.35	0.52
AHI	−0.004	0.01	0.99	0.98–1.02	0.68
Tsat90%	−0.016	0.01	0.98	0.90–1.01	0.15
Mitotic index	−0.047	0.03	0.95	0.90–1.01	0.12

*AHI, apnea–hypopnea index; Tsat90%, night-time spent with oxyhemoglobin saturation below 90%*.

A similar analysis was conducted for HIF-1α expression. In this case, the two variables introduced into the logistic regression analysis were the Breslow index, which was the best marker of melanoma aggressiveness, and the DI4% as a marker of IH. Table 8 shows that both variables were independently associated with higher expression of HIF-1α.

**Table 8:** Logistic regression analysis showing the independent relationships between the Breslow index and DI4% values and the presence of high expression levels of hypoxia inducible factor-1α in cutaneous melanoma tumors.

Variable	β coefficient	SE	Odds ratio	95% CI	*p*
Breslow index	0.24	0.068	1.28	1.18–1.46	**0.0001**
DI4%	0.33	0.098	1.03	1.01–1.06	**0.001**

*DI4%; desaturation index at 4%*.

*p < 0.05*.

## Discussion

This study shows that levels of HIF-1α expression in CM are independently associated with indicators of episodic hypoxia as derived from nocturnal polygraphic recordings in a large prospectively recruited cohort of CM patients. However, contrary to the significant associations between the patterns of HIF-1α expression and the severity of sleep-disordered breathing, VEGF expression was high in the vast majority of CM lesions, and did not manifest any significant relationship with polygraphically derived measurements, suggesting the absence of co-linearity between HIF-1α and VEGF expression in CM.

Before we discuss the potential implications of our findings, several methodological points deserve mention. First, this is the first prospective study concurrently evaluating one type of cancer, namely CM, and the presence of sleep-disordered breathing. Indeed, most of the existing literature to date has relied on retrospective databases, or alternatively, due to cohort size limitations, has not investigated a specific type of malignancy (28, 30, 35–41). The current study overcomes this major limitation, even if the methodological approach for assessment of sleep-disordered breathing consisted of overnight polygraphy rather than polysomnography, such that the role of disrupted sleep cannot be deduced (42). Of note, the prevalence of OSA and the distribution of severity categories of OSA in this cohort were similar to that reported in other population-based studies (43, 44), further attesting to the overall absence of bias in patient recruitment, which as indicated was guided by the diagnosis of CM. Second, to minimize any potential bias, we ascertained that there would be complete separation in the processing of the polygraphy scoring and the classification of the aggressiveness CM markers by the various expert panels. Finally, we implemented highly stringent and blinded HIF-1α and VEGF immunohistochemistry staining and cell counting procedures. However, we opted for a straightforward assessment of the expression of HIF-1α and VEGF to establish the potential validity of the *a priori* assumption, i.e., OSA is associated with increased hypoxia-related markers in CM tumors. In light of the current findings, future exploration of the major cell lineage subsets in which the presence of OSA induces the increased expression of HIF-1α would be of potential interest to the understanding of the dynamic underpinnings regulating tumor growth and metastatic potential. We should also remark that the number of CM patients with very high expression of HIF-1α was small (*n* = 16) and reflects the quartile-based categorical stratification approach undertaken here, as dictated by statistical considerations. Indeed, expansion of the number of staining intensity categories such as to offer more refined analyses would have required a much large cohort size to achieve adequate statistical power.

Indeed, previous work has indicated that HIF-1α plays pathophysiological roles in some of the previously identified morbid consequences of the disease. For example, the IH of OSA has been shown to contribute to liver fibrosis *via* recruitment of HIF-1α signaling (45). Similarly, targeting HIF-1α-related pathways may attenuate cardiovascular and metabolic consequences of IH (46–50). Thus, it is reasonable to assume that increased HIF-1α expression in the context of sleep-disordered breathing in our cohort would lead to increased HIF-1α expression in tissues in general and more specifically in the CM lesions, where its transcriptional activity could have fostered increased proliferation and other aggressiveness indicators (14–16). In contrast, the absence of any significant association between VEGF expression in the CM sections and correlates of nocturnal hypoxemia was surprising. Indeed, previous studies have shown that circulating levels of VEGF are increased in OSA (51–54), suggesting that similar patterns may be present in tissues. However, the presence of an unfavorable balance between VEGF and endothelial and vascular factors that may promote vascular injury has been suggested in OSA and could reduce the efficacy of the VEGF pro-angiogenic activity (55). Alternatively, chronic IH may attenuate rather promote the transcription of HIF-1α at the promoter level of its gene targets as recently shown (50), such that the major driver for increased VEGF expression in the tumors could be the intrinsic intra-tumoral hypoxia rather than the IH of OSA. Under such circumstances, it is also possible that the increased VEGF expression may not necessarily reflect the severity of OSA or of intra-tumoral hypoxia, and may be driven by alternative transcriptional regulators such as HIF-2 (21). Notwithstanding, the presence of independent associations between a prognostic indicator of CM (i.e., Breslow index), and a measure of OSA severity (i.e., DI4%) as explaining the variance in HIF-1α expression abundance suggest that the presence of OSA and its severity may contribute to the malignant characteristics of CM, and play a deleterious role in the outcomes of this highly prevalent tumor.

## Conclusion

In a large multicenter cohort of patients being diagnosed with CM, the expression of HIF-1α in the tumoral lesions is independently associated with nocturnal IH measures of sleep disordered breathing severity. These findings provide additional support to the evolving epidemiological and biological evidence whereby sleep apnea may play a deleterious role in cancer outcomes.

## Other Members of the Spanish Sleep Network

**Elidia Molina Herrera**, **Rosa M. García Martín**, Pathology Department. Hospital 12 de octubre, Madrid, Spain; **Maria Niveiro de Jaime**, Pathology Department, ISABIAL, Hospital Gral, Univ. Alicante, Alicante, Spain, Departamento Medicina Clínica. Univ. Miguel Hernandez, Elche, Spain; **Sara Moreno**, Dermatology Department, Hospital Universitario Arnau de Vilanova, University of Lleida, IRBLleida, Lleida, Spain; **Ferran Barbé Ilia**, Respiratory Department, Group of Translational Research in Respiratory Medicine, Hospital Universitari Arnau de Vilanova and Santa Maria, IRBLleida, Lleida, Catalonia, Spain, Centro de Investigación Biomédica en Red de Enfermedades Respiratorias (CIBERES), Madrid, Spain; **Manuel Sánchez de la Torre**, Respiratory Department, Group of Translational Research in Respiratory Medicine, Hospital Universitari Arnau de Vilanova and Santa Maria, IRBLleida, Lleida, Catalonia, Spain, Centro de Investigación Biomédica en Red de Enfermedades Respiratorias (CIBERES), Madrid, Spain; **Esther de Eusebio**, Dermatology Department, Hospital Universitario de Guadalajara, Spain; **Pedro Landete**, Pneumology Service, Hospital la Princesa, Madrid, Spain; **Manuel Moragón Gordon**, Dermatology Department, Hospital Universitario, San Juan de Alicante, Spain; **Eva Arias**, Respiratory Department, Hospital 12 de Octubre, Madrid, Spain; **Fernando Masa**, Respiratory Department, Hospital Universitario S. Pedro Alcántara, Cáceres, Spain, Centro de Investigación Biomédica en Red de Enfermedades Respiratorias (CIBERES), Madrid, Spain; **Carlos González Herrada**, Dermatology Department, Hospital Universitario de Getafe, Madrid, Spain; **Cristina Carrera**, Dermatology Department, Hospital Clinic-IDIBAPS, Barcelona, Spain; **Aida Muñoz Ferrer**, Respiratory Department, Hospital Germans Trials y Pujol, Barcelona, Spain; **Aram Boada**, Dermatology Department, Hospital Germans Trials y Pujol, Barcelona, Spain; **Ana Fortuna**, **Mercé Mayos**, Respiratory Department, Hospital Santa Creu i Sant Pau, Barcelona, Spain; **Jesús Gardeazabal García**, Dermatology Department, Hospital Universitario Cruces, Bilbao, Spain.

## Ethics Statement

The study was approved by the human ethics committees at the various participating sites, and written informed consent was obtained from each participant in accordance with the Declaration of Helsinki. The following human subject committtes provided approval for the study: Central Ethics Committee: Comité ético de investigación biomédica of Hospital Politécnico y Universitario La Fe de Valencia. Protocol # Cod 2012/1048 (includes subjects recruited at the hospitals La Fe, Manises y Instituto Valenciano de Oncologia). Comité ético de investigación clínica (CEIC) del Hospital Clinic de Barcelona. Protocol # 25042013. Comité ético de investigación clínica del Hospital General de Alicante. Agencia Valenciana de Salud. Protocol # 2013/030. Comité ético de investigación clínica del Hospital San Juan de Alicante. Agencia Valenciana de Salud. Código 12/304. Comité ético de investigación clínica y ensayos clínicos de Getafe. Protocol # A03-13. Comité ético de investigación clínica y ensayos clínicos de Guadalajara (SESCAM). Protocol # 19032013. Comitè d’Etica de la investigaciò del Hospital Germans Trials i Pujol. Protocol # PI13-074. Comitè d’Etica de la investigaciò del Hospital Santa Creu i Sant Pau. No protocol #. Comitè d’Etica de la investigaciò (CEIC) del Hospital Arnau de Vilanova de Lleida. Protocol # CEIC-1154. Comité ético de investigación clínica y ensayos clínicos de la Comunidad autónoma del País Vasco. Protocol # PI2014046. Informe del Comité local de ensatos clínicos. Valme. Protocol # 301012. Comité ético de investigación clínica y ensayos clínicos de Cáceres. No protocol #.

## Author Contributions

IA, MM-G, FC-R, and DG participated in the conceptual framework of the project; MM-G., FC-R, ER-F, JR-P, EN, AM-C, LH-B, JR, EV, AS-T, JA-C, JM, AP-G, VC-N, IC-P, JC-P, TD-C, OM, and JD-A recruited patients and collected data and/or samples; MM-G, DG, IA, MM-G, FC-R, and RF interpreted data and drafted the manuscript. All authors approved the final version of the manuscript.

## Conflict of Interest Statement

The authors declare that the research was conducted in the absence of any commercial or financial relationships that could be construed as a potential conflict of interest. The handling Editor is currently co-organizing a Research Topic with one of the authors IA and confirms the absence of any other collaboration.
